# Examining concordance of sexual-related factors and PrEP eligibility with HIV risk perception among adolescent girls and young women: cross-sectional insights from DREAMS sites in Kenya, Malawi, and Zambia

**DOI:** 10.1186/s12889-024-20276-4

**Published:** 2024-10-12

**Authors:** Craig J. Heck, Domonique M. Reed, Jerry Okal, Effie Chipeta, Michael Mbizvo, Sanyukta Mathur

**Affiliations:** 1 Department of Epidemiology, Columbia University Mailman School of Public Health, New York, NY USA; 2 Division of Infectious Diseases, Department of Medicine, Columbia University Irving Medical Center, New York, NY USA; 3 Population Council, Nairobi, Kenya; 4 Centre for Reproductive Health, Kamuzu University of Health Sciences, Blantyre, Southern Region Malawi; 5 Population Council, Lusaka, Zambia; 6 Population Council, Washington, D.C USA

**Keywords:** HIV risk perception, HIV prevention, Pre-exposure prophylaxis, Adolescent girls and young women, DREAMS, Eastern and Southern Africa

## Abstract

**Background:**

HIV risk perception is an important cognition for prevention, theoretically engendering service-seeking and risk-reduction behaviors, but its composition remains poorly understood. We examined country-specific correlates of self-appraised HIV exposure risk among sexually active adolescent girls and young women (AGYW, aged 15–24 years) without HIV in Kenya, Malawi, and Zambia. We also explored overlaps between self-appraised HIV exposure risk and pre-exposure prophylaxis (PrEP) eligibility to identify engagement opportunities.

**Methods:**

We analyzed cross-sectional data (2016/2017) to estimate sexual-related correlates of self-appraised HIV exposure risk (likely vs. not, temporally framed as “ever”) using log-Poisson models with robust standard errors. For sexual-related factors with an unadjusted *p* ≤ 0.10, individual adjusted models were fitted, controlling for sociodemographic and cognitive factors with an unadjusted *p* ≤ 0.10. PrEP eligibility was defined using national guidelines; since conditional criteria are in Malawi’s (age-disparate sex + ever-pregnant) and Zambia’s (multiple partners + condomless sex) guidelines, we also assessed PrEP eligibility after decoupling these factors.

**Results:**

Few AGYW reported likely HIV exposures (Kenya [*N* = 746]: 15.7%, Malawi [*N* = 1348]: 46.2%, Zambia [*N* = 349]: 9.5%) despite ubiquitous HIV risk (98.7%, 99.8%, and 98.9% of Kenyan, Malawian, and Zambian AGYW reported ≥ 1 sexual-related factor). However, the adjusted models found some actual-perceived risk concordance. Positive correlates of self-appraised likely HIV exposures included partner(s)’ likely HIV exposure (all countries); partner(s)’ unknown HIV status and other partners (Kenya, Malawi); STI symptoms and partner(s) living outside the community (Kenya); non-partner sexual violence (Zambia); and transactional sex, multiple partners, pre-coital alcohol use, and physical/sexual intimate partner violence (Malawi). Per national guidelines, PrEP eligibility criteria differentially identified HIV risk (Kenya: 93.6%, Malawi: 53.3%, Zambia: 44.6%), and self-appraised likely HIV exposures were low among PrEP-eligible AGYW (Kenya: 16.5%, Malawi: 48.5%, Zambia: 18.8%). Decoupling Malawi’s and Zambia’s conditional PrEP criteria could increase risk identification to > 85% and potential engagement by ~ 70% and ~ 30%, respectively.

**Conclusions:**

AGYW's HIV risk perceptions were mostly influenced by factors beyond their locus of control. Conditional PrEP eligibility criteria may inhibit AGYW’s access and uptake in some settings: countries should consider decoupling these factors to minimize barriers. Intersections between autonomy, behaviors, and perceptions among AGYW in gender-inequitable settings warrants further investigation.

**Supplementary Information:**

The online version contains supplementary material available at 10.1186/s12889-024-20276-4.

## Introduction

The United Nations has committed to ending HIV/AIDS by 2030; to increase the likelihood of achieving this goal, interim 2025 targets were set at ≤ 370,000 annual global seroconversions [1, 2]. Given 1.3 million people acquired HIV in 2022, epidemic control is unlikely without swift, marked intervention in high-incidence settings, like Eastern and South Africa (ESA), where approximately 40% of global seroconversions occur [3]. Adolescent girls and young women (AGYW, aged 15–24 years) account for 26% of seroconversions in ESA, highlighting reducing their incidence is a priority for epidemic control efforts [4].

The expanding HIV prevention toolkit provides AGYW with new options to reduce their HIV risk. In 2015, once-daily oral pre-exposure prophylaxis (PrEP) became the first prevention method AGYW could use without partner cooperation, but initiation, adherence, and continuation remains low due to pill burden, side effect concerns, and stigma [5–9]. Long-acting PrEP (LA-PrEP) methods—like monthly Dapivirine vaginal ring; bimonthly injectable Cabotegravir; and based on recent trial data, twice-yearly injectable Lenacapavir—may circumvent these issues by offering AGYW more convenient, discreet protection modalities [10–12]. Increasing choice may also improve PrEP outcomes by enabling AGYW to choose a method that aligns with their preferences and circumstances [13, 14]. With the imminent availability of LA-PrEP methods, and others on the horizon, AGYW’s effective use of HIV prevention, particularly PrEP, is imperative for epidemic control efforts [15].

Despite their heightened risk for HIV acquisition, AGYW do not reliably use HIV prevention services and methods [16]. Lack of perceived need may potentially explain this disconnect. Social-behavioral theories hypothesize risk perception is a key antecedent to health promotion behaviors: individuals who perceive themselves at risk for an adverse outcome will be motivated toward risk-reduction behaviors [17–19]. These theorized relationships are problematized by AGYW’s HIV-associated behaviors contradicting their low HIV risk perception, leading to oral PrEP non-initiation and discontinuation, inconsistent condom use, and infrequent HIV testing [20–23]. Discordance between actual and perceived HIV risk is a pervasive issue that has been observed in other populations and settings [24–26]. Greater understanding of AGYW’s HIV risk perception is vital to increasing their access of HIV prevention services and maximizing the preventative potential of current and future prevention methods.

Improving AGYW’s HIV prevention outcomes necessitates understanding of the behaviors, circumstances, and experiences that influence how they perceive their risk of HIV acquisition. However, the underpinnings of AGYW’s HIV risk perception are rarely examined, highlighting its formulation is poorly understood. Illuminating these factors could inform activities and interventions to improve AGYW’s access, initiation, and effective use of HIV prevention services and methods, including PrEP [18, 19, 27]. To address this knowledge gap, we examined country-specific correlates of HIV risk perception among AGYW in Kenya, Malawi, and Zambia. We also explored overlaps between PrEP eligibility and HIV risk perception to identify opportunities and improvements for PrEP engagement.

## Methods

### Study setting and participants

This secondary analysis used cross-sectional data collected from AGYW residing in DREAMS (Determined, Resilient, Empowered, AIDS-free, Mentored and Safe) study sites in urban/peri-urban Kenya (2016–2017), rural Malawi (2017), and urban Zambia (2016–2017) during DREAMS’ pre-intervention phase. Briefly, DREAMS was a multi-component and -sectoral partnership to reduce AGYW’s HIV risk by implementing a suite of interventions targeting AGYW, families, partners, and communities [28]. The sampling and survey methods are described elsewhere; in summary, using beneficiary rosters (Kenya, Malawi) and censuses (Zambia) created by local implementing partners, potential respondents were identified using age-stratified random sampling [29]. Eligible participants included AGYW who agreed to participate and stay within the study area in the next year; for Malawi, out-of-school status was another criterion. Kenya’s and Zambia’s samples included DREAMS and non-DREAMS enrollees; Malawi’s sample included only DREAMS enrollees. Participants completed a self-administered questionnaire capturing HIV-related knowledge, attitudes, and practices.

To focus on HIV prevention, the samples included AGYW who self-reported being HIV negative and having sex in the last 12 months (sexually active). In Zambia, the sample comprised those currently having sex at the time of the interview due to questionnaire skip logic.

### Measures

Supplemental Material 1 presents the definitions for all study measures.

### Dependent variable

We measured HIV risk perception using self-appraised likelihood of HIV exposure, "how likely it is that you have been exposed to HIV?", a question asked after all HIV-related risk modules, facilitating a retrospective assessment. We operationalized response categories as Likely (very likely, somewhat likely) vs. Unlikely (unlikely, not at all), excluding participants with missing or “don’t know” responses [30–32].

### Sociodemographic factors

To identify variables for multivariable control, we explored sociodemographic factors, encompassing age group (adolescent girls, aged 15–19 years [AG] vs. young women, aged 20–24 years [YW]); ever-pregnant, orphanhood, out-of-school, employment, marital, and socio-economic status (SES); hunger; location; and DREAMS enrollee status.

### Cognitive factors

We assessed anxiety/depression symptoms and comprehensive HIV knowledge because they can vary risk perception [33, 34].

### Sexual-related factors

First, we examined HIV-associated behaviors, including inconsistent condom use, STI symptoms, age-disparate and inter-generational sex, transactional sex, multiple sexual partnerships, and pre-coital alcohol use. Next, we assessed relationship characteristics: partner(s)’ 1) community residence, 2) other partners, 3) HIV status, and 4) likely HIV exposure. Finally, we investigated respondents' survivorship of physical and sexual intimate partner violence (IPV) and non-partner sexual violence.

### PrEP eligibility

PrEP eligibility was measured using criteria in each country’s national guidelines. Below, we outline the criteria for each country, followed by analytic definitions in parentheses.

Kenya’s criteria included multiple partners (multiple partners), condomless sex (inconsistent condom use), partners’ unknown HIV status (does not know partner[s] HIV status), any partners at risk of HIV (partner[s] likely exposed to HIV), STI diagnosis (STI symptoms in last 6 months), received money/material support for sex (transactional sex), survived forced sex or physical assault (survived non-partner sexual violence or physical or sexual IPV), and history of sex while under the influence of drugs/alcohol (alcohol use) [35, 36].

For Malawi, eligibility criteria comprised having an STI in the last 6 months (STI symptoms in last 6 months), giving/receiving monetary incentives for sex (transactional sex), and having an age-disparate partner and ever being pregnant (positive indications for both ever-pregnant and age-disparate sex, as defined by national guidelines) [37, 38].

In Zambia, criteria encompassed sex with multiple partners without condoms (positive indications for both multiple partners and inconsistent condom use, as defined by national guidelines), partner has HIV/at substantial risk of acquiring HIV (partner[s] likely exposed to HIV), history of STIs (STI symptoms in last 6 months), and prior post-exposure prophylaxis (PEP) use (ever used PEP) [39].

We could not include sero-different partnership dynamics (Kenya, Malawi, Zambia), sharing injection materials (Kenya, Zambia), or PEP use (Kenya) due to questionnaire limitations.

We operationalized PrEP eligibility as a binary measure (≥ 1 criteria versus None). For Malawi and Zambia, we also explored eligibility by decoupling their conditional criteria (e.g., Malawi: age-disparate sex and ever-pregnant, Zambia: multiple partners and inconsistent condom use).

### Statistical analyses

We examined country-specific correlates of likely HIV exposure, the dependent variable, using prevalence ratios (log-Poisson models, robust standard errors) generated with R (v.4.4.0) [40–44]. We fitted an adjusted model for each sexual-related factor with an unadjusted *p* ≤ 0.10; sociodemographic and cognitive control variables were selected using α = 0.10. Sexual-related factors with an adjusted *p* ≤ 0.05 were considered correlates.

We descriptively explored intersections between HIV risk, PrEP eligibility, and risk perception by assessing PrEP eligibility among AGYW with ≥ 1 sexual-related risk factor and describing the proportion of AGYW reporting likely HIV exposures by PrEP eligibility status.

## Results

Overall, 5364 eligible AGYW in Kenya (*n* = 1777), Malawi (*n* = 1672), and Zambia (*n* = 1915) participated in the survey. After excluding respondents due to sexual inactivity, HIV status, and missing or don’t know responses for the outcome, Kenya’s, Malawi’s, and Zambia’s analytic samples included 746, 1348, and 349 AGYW, respectively (Supplemental Material 2).

### Descriptive statistics of Kenya, Malawi, and Zambia samples

Across countries, most respondents were YW (Kenya: 72.7%, Malawi: 72.3%, Zambia: 79.4%) who had ever been pregnant (Kenya: 74.7%, Malawi: 94.8%, Zambia: 67.3%; Tables 1, 2 and 3). In-school status (Kenya: 79.4%, Zambia: 72.2%) and unemployment (Kenya: 59.7%, Zambia: 51.9%) were common in Kenya and Zambia, and most Kenyan (60.6%) and Malawian (86.9%) respondents were married. Low SES status (Kenya: 43.3%, Malawi: 30.0%, Zambia: 31.5%), food insecurity (Kenya: 37.1%, Malawi: 35.2%, Zambia: 18.9%), anxiety/depression symptoms (Kenya: 21.4%, Malawi: 26.6%, Zambia: 36.7%), and incomplete HIV knowledge (Kenya: 42.8%, Malawi: 49.3%, Zambia: 40.1%) were relatively uncommon.

**Table 1:** Correlates of risk perception among sexually active AGYW in Kenya

**Characteristic**	**Overall *N* = 746**	**HIV exposure**		**PR [95% CI]**	***p*-value**	**APR [95% CI]**
		**Unlikely *N* = 629**	**Likely *N* = 117**			
***Sociodemographic factors***						
**Age (categorical)**						
*15–19 years*	27.3% (204/746)	88.2% (180/204)	11.8% (24/204)	—		
*20–24 years*	72.7% (542/746)	82.8% (449/542)	17.2% (93/542)	1.46 [0.96–2.22]	0.077	
**Ever Pregnant**						
*No*	25.3% (189/746)	84.7% (160/189)	15.3% (29/189)	—		
*Yes*	74.7% (557/746)	84.2% (469/557)	15.8% (88/557)	1.03 [0.70–1.51]	0.882	
**Orphaned**						
*No*	40.2% (300/746)	85.0% (255/300)	15.0% (45/300)	—		
*Yes*	59.8% (446/746)	83.9% (374/446)	16.1% (72/446)	1.08 [0.76–1.52]	0.674	
**Out of School**						
*No*	79.4% (592/746)	85.5% (506/592)	14.5% (86/592)	—		
*Yes*	20.6% (154/746)	79.9% (123/154)	20.1% (31/154)	1.39 [0.96–2.01]	0.084	
**Currently Employed**						
*No*	59.7% (445/746)	85.8% (382/445)	14.2% (63/445)	—		
*Yes*	40.3% (301/746)	82.1% (247/301)	17.9% (54/301)	1.27 [0.91–1.77]	0.163	
**Married**						
*No*	39.4% (294/746)	84.0% (247/294)	16.0% (47/294)	—		
*Yes*	60.6% (452/746)	84.5% (382/452)	15.5% (70/452)	0.97 [0.69–1.36]	0.854	
**Socioeconomic Status**						
*Low (0–2 items)*	43.3% (323/746)	82.4% (266/323)	17.6% (57/323)	—		
*Medium/High (3–5 items)*	56.7% (423/746)	85.8% (363/423)	14.2% (60/423)	0.80 [0.58–1.12]	0.198	
**Hungry in last 4 weeks**						
*No*	62.9% (469/746)	87.8% (412/469)	12.2% (57/469)	—		
*Yes*	37.1% (277/746)	78.3% (217/277)	21.7% (60/277)	1.78 [1.28–2.48]	**< 0.001**	
**Location**						
*Site 1*	52.1% (389/746)	83.5% (325/389)	16.5% (64/389)	—		
*Site 2*	47.9% (357/746)	85.2% (304/357)	14.8% (53/357)	0.90 [0.65–1.26]	0.547	
**DREAMS enrollee**						
*No*	55.6% (415/746)	87.2% (362/415)	12.8% (53/415)	—		
*Yes*	44.4% (331/746)	80.7% (267/331)	19.3% (64/331)	1.51 [1.08–2.11]	**0.015**	
***Cognitive factors***						
**Self-reported Anxiety & Depression Symptoms**						
*Normal*	78.6% (586/746)	88.4% (518/586)	11.6% (68/586)	—		
*Mild/Moderate/Severe*	21.4% (160/746)	69.4% (111/160)	30.6% (49/160)	2.64 [1.91–3.65]	**< 0.001**	
**Comprehensive HIV Knowledge**						
*No*	42.8% (319/746)	84.3% (269/319)	15.7% (50/319)	—		
*Yes*	57.2% (427/746)	84.3% (360/427)	15.7% (67/427)	1.00 [0.72–1.40]	0.995	
***Sexual Behaviors, Experiences, and/or Circumstances***						
**At risk for HIV (> = 1 sex-related factor)**						
*No*	1.3% (10/746)	100.0% (10/10)	0.0% (0/10)	‡		
*Yes*	98.7% (736/746)	84.1% (619/736)	15.9% (117/736)	‡	‡	
**Inconsistent condom use**^**a**^						
*No*	24.7% (184/746)	83.2% (153/184)	16.8% (31/184)	—		
*Yes*	75.3% (562/746)	84.7% (476/562)	15.3% (86/562)	0.91 [0.62–1.32]	0.615	
**STI symptoms**^**a**^						
*No*	76.3% (569/746)	86.6% (493/569)	13.4% (76/569)	—		—
*Yes*	23.7% (177/746)	76.8% (136/177)	23.2% (41/177)	1.73 [1.23–2.44]	**0.002**	**1.44 [1.03–2.01]**
**Age-disparate sex**						
*No*	56.2% (407/724)	86.0% (350/407)	14.0% (57/407)	—		
*Yes*	43.8% (317/724)	83.6% (265/317)	16.4% (52/317)	1.17 [0.83–1.66]	0.370	
**Inter-generational sex**						
*No*	89.4% (647/724)	85.5% (553/647)	14.5% (94/647)	—		
*Yes*	10.6% (77/724)	80.5% (62/77)	19.5% (15/77)	1.34 [0.82–2.19]	0.242	
**Transactional sex**^**a**^						
*No*	94.5% (704/745)	84.9% (598/704)	15.1% (106/704)	—		—
*Yes*	5.5% (41/745)	73.2% (30/41)	26.8% (11/41)	1.78 [1.04–3.04]	**0.034**	1.53 [0.90–2.59]
**Multiple sexual partners**^**a**^						
*No*	82.6% (616/746)	85.4% (526/616)	14.6% (90/616)	—		—
*Yes*	17.4% (130/746)	79.2% (103/130)	20.8% (27/130)	1.42 [0.97–2.09]	0.074	1.43 [0.98–2.09]
**Alcohol use before sex**^**a**^						
*No*	90.9% (678/746)	85.1% (577/678)	14.9% (101/678)	—		—
*Yes*	9.1% (68/746)	76.5% (52/68)	23.5% (16/68)	1.58 [0.99–2.51]	0.054	1.34 [0.84–2.12]
**Partner(s) does not live in community**						
*No*	62.6% (467/746)	86.7% (405/467)	13.3% (62/467)	—		—
*Yes*	37.4% (279/746)	80.3% (224/279)	19.7% (55/279)	1.48 [1.07–2.07]	**0.019**	**1.40 [1.00–1.95]**
**Partner(s) had other partners**						
*No*	55.0% (410/746)	88.5% (363/410)	11.5% (47/410)	—		—
*Don't know*	30.4% (227/746)	82.8% (188/227)	17.2% (39/227)	1.50 [1.01–2.22]	**0.043**	1.42 [0.96–2.10]
*Yes*	14.6% (109/746)	71.6% (78/109)	28.4% (31/109)	2.48 [1.66–3.71]	**< 0.001**	**2.23 [1.51–3.28]**
**Does not know partner(s) HIV status†**						
*No*	87.1% (650/746)	86.0% (559/650)	14.0% (91/650)	—		—
*Yes*	12.9% (96/746)	72.9% (70/96)	27.1% (26/96)	1.93 [1.32–2.83]	**< 0.001**	**1.81 [1.24–2.63]**
**Partner(s) likely exposed to HIV**^**a**^						
*No*	69.6% (519/746)	92.5% (480/519)	7.5% (39/519)	—		—
*Don't know*	14.5% (108/746)	86.1% (93/108)	13.9% (15/108)	1.85 [1.06–3.23]	**0.031**	**1.82 [1.06–3.14]**
*Yes*	16.0% (119/746)	47.1% (56/119)	52.9% (63/119)	7.05 [4.98–9.96]	**< 0.001**	**5.77 [3.95–8.43]**
**Non-partner sexual violence survivorship**^**a**^						
*No*	77.1% (575/746)	86.3% (496/575)	13.7% (79/575)	—		—
*Yes*	22.9% (171/746)	77.8% (133/171)	22.2% (38/171)	1.62 [1.14–2.29]	**0.007**	1.29 [0.92–1.80]
**Physical and/or sexual violence survivorship**^**a**^						
*No*	51.6% (385/746)	88.6% (341/385)	11.4% (44/385)	—		—
*Yes*	48.4% (361/746)	79.8% (288/361)	20.2% (73/361)	1.77 [1.25–2.50]	**0.001**	1.36 [0.96–1.94]

Bolded aPR [95% CI] indicates *p* ≤ 0.05

*PR* Prevalence Ratio, *CI* Confidence Interval, *APR* Adjusted Prevalence Ratio

^a^Oral pre-exposure prophylaxis eligibility criterion

^‡^Regression model not fitted due to small cell sizes

Nearly all respondents (Kenya: 98.7%, Malawi: 99.8%, Zambia: 98.9%) reported at least one sexual-related HIV-associated factor. Inconsistent condom use was the most prevalent HIV-associated behavior in all countries (Kenya: 75.3%, Malawi: 93.9%, Zambia: 74.8%). Violence survivorship (Kenya: 48.4%, Malawi: 37.5%, Zambia: 45.3%) and age-disparate sex (Kenya: 43.8%, Malawi: 33.4%, Zambia: 57.8%) were also prevalent. Believing partner(s) were likely exposed to HIV was particularly common in Malawi (Kenya: 16.0%, Malawi: 58.1%, Zambia: 8.3%).

### Sexual-related correlates of likely HIV exposures

#### Kenya

Table 1 presents the Kenya-specific findings. In total, 15.6% (117/746) of Kenyan AGYW reported a likely HIV exposure. Based on the unadjusted analyses, the adjusted models controlled for categorical age, out-of-school status, food insecurity, DREAMS enrollment, and anxiety/depression symptoms.

After controlling for these sociodemographic and cognitive factors, correlates of likely HIV exposures included STI symptoms (Adjusted Prevalence Ratio [APR] = 1.44 [1.03–2.01]) and partner(s)’ non-community residence (APR = 1.40 [1.00–1.95]), other partners (APR = 2.23 [1.51–3.28]), unknown status (APR = 1.81 [1.24–2.63]), and likely HIV exposure (Don’t’ know: APR = 1.82 [1.06–3.14], Yes: APR = 5.77 [3.95–8.43], ref: No).

#### Malawi

Table 2 showcases Malawi’s associations. Overall, 46.2% (623/1348) of Malawian AGYW stated they likely had an HIV exposure. Sociodemographic and cognitive control variables comprised employment, food insecurity, location, and anxiety/depression symptoms.

**Table 2:** Correlates of risk perception among sexually active AGYW in Malawi

**Characteristic**	**Overall *N* = 1348**	**HIV exposure**		**PR [95% CI]**	***p*-value**	**APR [95% CI]**
		**Unlikely *N* = 725**	**Likely *N* = 623**			
***Sociodemographic factors***						
**Age (categorical)**						
*15–19 years*	27.7% (373/1,346)	56.6% (211/373)	43.4% (162/373)	—		
*20–24 years*	72.3% (973/1,346)	52.6% (512/973)	47.4% (461/973)	1.09 [0.95–1.25]	0.201	
**Ever Pregnant**						
*No*	5.2% (70/1,348)	55.7% (39/70)	44.3% (31/70)	—		
*Yes*	94.8% (1,278/1,348)	53.7% (686/1,278)	46.3% (592/1,278)	1.05 [0.80–1.37]	0.743	
**Orphaned**						
*No*	64.7% (872/1,348)	53.8% (469/872)	46.2% (403/872)	—		
*Yes*	35.3% (476/1,348)	53.8% (256/476)	46.2% (220/476)	1.00 [0.89–1.13]	> 0.999	
**Out of School**						
*No*	0.0% (0/1381)	-	-	-		
*Yes*	100.0% (1381/1381)	-	-	-	-	
**Currently Employed**						
*No*	9.4% (127/1,348)	70.1% (89/127)	29.9% (38/127)	—		
*Yes*	90.6% (1,221/1,348)	52.1% (636/1,221)	47.9% (585/1,221)	1.60 [1.22–2.10]	**< 0.001**	
**Married**						
*No*	13.1% (176/1,348)	59.1% (104/176)	40.9% (72/176)	—		
*Yes*	86.9% (1,172/1,348)	53.0% (621/1,172)	47.0% (551/1,172)	1.15 [0.95–1.39]	0.146	
**Socioeconmic Status**						
*Low (0–1 items)*	30.0% (404/1,348)	55.2% (223/404)	44.8% (181/404)	—		
*Medium (2 items)*	41.2% (555/1,348)	51.2% (284/555)	48.8% (271/555)	1.09 [0.95–1.25]	0.221	
*High (3–5 items)*	28.9% (389/1,348)	56.0% (218/389)	44.0% (171/389)	0.98 [0.84–1.15]	0.811	
**Hungry in last 4 weeks**						
*No*	64.8% (873/1,348)	60.4% (527/873)	39.6% (346/873)	—		
*Yes*	35.2% (475/1,348)	41.7% (198/475)	58.3% (277/475)	1.47 [1.32–1.65]	**< 0.001**	
**Location**						
*Site 1*	31.4% (423/1,348)	60.5% (256/423)	39.5% (167/423)	—		
*Site 2*	68.6% (925/1,348)	50.7% (469/925)	49.3% (456/925)	1.25 [1.09–1.43]	**0.001**	
**DREAMS enrollee**						
*No*	0.0% (0/1381)	-	-	-		
*Yes*	100.0% (1381/1381)	-	-	-	-	
***Cognitive factors***						
**Self-reported Anxiety & Depression Symptoms**						
*Normal*	73.4% (989/1,348)	57.9% (573/989)	42.1% (416/989)	—		
*Mild/Moderate/Severe*	26.6% (359/1,348)	42.3% (152/359)	57.7% (207/359)	1.37 [1.22–1.54]	**< 0.001**	
**Comprehensive HIV Knowledge**						
*No*	49.3% (665/1,348)	52.9% (352/665)	47.1% (313/665)	—		
*Yes*	50.7% (683/1,348)	54.6% (373/683)	45.4% (310/683)	0.96 [0.86–1.08]	0.536	
***Sexual Behaviors, Experiences, and/or Circumstances***						
**At risk for HIV (> = 1 sex-related factor)**						
*No*	0.2% (3/1,348)	66.7% (2/3)	33.3% (1/3)	‡		
*Yes*	99.8% (1,345/1,348)	53.8% (723/1,345)	46.2% (622/1,345)	‡	‡	
**Inconsistent condom use**						
*No*	6.1% (82/1,348)	51.2% (42/82)	48.8% (40/82)	—		
*Yes*	93.9% (1,266/1,348)	53.9% (683/1,266)	46.1% (583/1,266)	0.94 [0.75–1.19]	0.623	
**STI symptoms**^**a**^						
*No*	68.6% (925/1,348)	56.6% (524/925)	43.4% (401/925)	—		—
*Yes*	31.4% (423/1,348)	47.5% (201/423)	52.5% (222/423)	1.21 [1.08–1.36]	**0.001**	1.08 [0.96–1.21]
**Age-disparate sex**						
*No*	66.6% (898/1,348)	54.6% (490/898)	45.4% (408/898)	—		
*Yes*	33.4% (450/1,348)	52.2% (235/450)	47.8% (215/450)	1.05 [0.93–1.19]	0.412	
**Age-disparate sex & Ever-pregnant**^**a**^						
*No*	67.5% (910/1,348)	54.4% (495/910)	45.6% (415/910)	—		
*Yes*	32.5% (438/1,348)	52.5% (230/438)	47.5% (208/438)	1.04 [0.92–1.18]	0.513	
**Inter-generational sex**						
*No*	94.2% (1,270/1,348)	53.7% (682/1,270)	46.3% (588/1,270)	—		
*Yes*	5.8% (78/1,348)	55.1% (43/78)	44.9% (35/78)	0.97 [0.75–1.25]	0.808	
**Transactional sex**^**a**^						
*No*	96.7% (1,304/1,348)	54.5% (711/1,304)	45.5% (593/1,304)	—		—
*Yes*	3.3% (44/1,348)	31.8% (14/44)	68.2% (30/44)	1.50 [1.21–1.85]	**< 0.001**	**1.37 [1.13–1.66]**
**Multiple sexual partners**						
*No*	91.6% (1,235/1,348)	54.7% (675/1,235)	45.3% (560/1,235)	—		—
*Yes*	8.4% (113/1,348)	44.2% (50/113)	55.8% (63/113)	1.23 [1.03–1.47]	**0.021**	**1.21 [1.02–1.44]**
**Alcohol use before sex**						
*No*	90.9% (1,225/1,348)	54.7% (670/1,225)	45.3% (555/1,225)	—		—
*Yes*	9.1% (123/1,348)	44.7% (55/123)	55.3% (68/123)	1.22 [1.03–1.45]	**0.022**	**1.22 [1.03–1.45]**
**Partner(s) does not live in community**						
*No*	77.4% (1,043/1,348)	54.0% (563/1,043)	46.0% (480/1,043)	—		
*Yes*	22.6% (305/1,348)	53.1% (162/305)	46.9% (143/305)	1.02 [0.89–1.17]	0.789	
**Partner(s) had other partners**						
*No*	46.7% (629/1,348)	61.7% (388/629)	38.3% (241/629)	—		—
*Don't know*	34.9% (471/1,348)	50.1% (236/471)	49.9% (235/471)	1.30 [1.14–1.49]	**< 0.001**	**1.29 [1.13–1.47]**
*Yes*	18.4% (248/1,348)	40.7% (101/248)	59.3% (147/248)	1.55 [1.34–1.79]	**< 0.001**	**1.43 [1.24–1.65]**
**Does not know partner(s) HIV status**						
*No*	82.9% (1,117/1,348)	55.1% (615/1,117)	44.9% (502/1,117)	—		—
*Yes*	17.1% (231/1,348)	47.6% (110/231)	52.4% (121/231)	1.17 [1.01–1.34]	**0.031**	**1.16 [1.02–1.33]**
**Partner(s) likely exposed to HIV**						
*No*	38.3% (516/1,348)	78.9% (407/516)	21.1% (109/516)	—		—
*Don't know*	3.6% (49/1,348)	71.4% (35/49)	28.6% (14/49)	1.35 [0.84–2.17]	0.211	1.25 [0.78–2.01]
*Yes*	58.1% (783/1,348)	36.1% (283/783)	63.9% (500/783)	3.02 [2.54–3.60]	**< 0.001**	**2.86 [2.40–3.41]**
**Physical intimate partner violence survivorship**						
*No*	83.5% (1,125/1,348)	55.6% (625/1,125)	44.4% (500/1,125)	—		—
*Yes*	16.5% (223/1,348)	44.8% (100/223)	55.2% (123/223)	1.24 [1.08–1.42]	**0.002**	**1.16 [1.01–1.33]**
**Sexual intimate partner violence survivorship**						
*No*	82.1% (1,107/1,348)	59.3% (656/1,107)	40.7% (451/1,107)	—		—
*Yes*	17.9% (241/1,348)	28.6% (69/241)	71.4% (172/241)	1.75 [1.57–1.95]	**< 0.001**	**1.56 [1.39–1.75]**
**Non-partner sexual violence survivorship**						
*No*	91.7% (1,236/1,348)	53.9% (666/1,236)	46.1% (570/1,236)	—		
*Yes*	8.3% (112/1,348)	52.7% (59/112)	47.3% (53/112)	1.03 [0.84–1.26]	0.805	
**Physical and/or sexual violence survivorship**						
*No*	62.5% (842/1,348)	60.6% (510/842)	39.4% (332/842)	—		—
*Yes*	37.5% (506/1,348)	42.5% (215/506)	57.5% (291/506)	1.46 [1.30–1.63]	**< 0.001**	**1.31 [1.16–1.47]**

Bolded aPR [95% CI] indicates *p* ≤ 0.05

*PR* Prevalence Ratio, *CI* Confidence Interval, *APR* Adjusted Prevalence Ratio

^a^Oral pre-exposure prophylaxis eligibility criterion

^‡^Regression model not fitted due to small cell sizes

After adjustment, transactional sex (APR = 1.37 [1.13–1.66]); multiple sex partners (APR = 1.21 [1.02–1.44]); alcohol use (APR = 1.22 [1.03–1.45]); partner(s)’ other partners (Don’t’ know: APR = 1.29 [1.13–1.47], Yes: APR = 1.43 [1.24–1.65], ref: No), unknown status (APR = 1.16 [1.02–1.33]), and likely HIV exposure (Yes: APR = 2.86 [2.40–3.41], ref: No); and physical (APR = 1.16 [1.01–1.33]) and sexual (APR = 1.56 [1.39–1.75]) IPV emerged as correlates.

#### Zambia

Table 3 highlights Zambia’s relationships. Only 9.5% (33/349) of Zambian AGYW reported a likely HIV exposure. After controlling for pregnancy history, out-of-school and marital status, location, and DREAMS enrollment, partner(s)’ likely HIV exposure (Don’t know: APR = 6.22 [2.71–14.2], Yes: APR = 10.70 [5.07–22.70],ref: No) and non-partner sexual violence (APR = 2.04 [1.04–4.00]) were correlates.

**Table 3:** Correlates of risk perception among currently sexually active AGYW in Zambia

**Characteristic**	**Overall *N* = 349**	**HIV exposure**		**PR [95% CI]**	***p*-value**	**APR [95% CI]**
		**Unlikely *N* = 316**	**Likely *N* = 33**			
***Sociodemographic factors***						
**Age (categorical)**						
*15–19 years*	20.6% (72/349)	87.5% (63/72)	12.5% (9/72)	—		
*20–24 years*	79.4% (277/349)	91.3% (253/277)	8.7% (24/277)	0.69 [0.34–1.43]	0.319	
**Ever Pregnant**						
*No*	32.7% (114/349)	82.5% (94/114)	17.5% (20/114)	—		
*Yes*	67.3% (235/349)	94.5% (222/235)	5.5% (13/235)	0.32 [0.16–0.61]	**< 0.001**	
**Orphaned**						
*No*	49.6% (173/349)	89.0% (154/173)	11.0% (19/173)	—		
*Yes*	50.4% (176/349)	92.0% (162/176)	8.0% (14/176)	0.72 [0.38–1.40]	0.336	
**Out of School**						
*No*	72.2% (252/349)	94.0% (237/252)	6.0% (15/252)	—		
*Yes*	27.8% (97/349)	81.4% (79/97)	18.6% (18/97)	3.12 [1.64–5.94]	**< 0.001**	
**Currently Employed**						
*No*	51.9% (181/349)	89.5% (162/181)	10.5% (19/181)	—		
*Yes*	48.1% (168/349)	91.7% (154/168)	8.3% (14/168)	0.79 [0.41–1.53]	0.491	
**Married**						
*No*	54.2% (189/349)	86.8% (164/189)	13.2% (25/189)	—		
*Yes*	45.8% (160/349)	95.0% (152/160)	5.0% (8/160)	0.38 [0.18–0.81]	**0.013**	
**Socioeconmic Status**						
*Low (0–3 items)*	31.5% (110/349)	93.6% (103/110)	6.4% (7/110)	—		
*Medium (4 items)*	37.0% (129/349)	88.4% (114/129)	11.6% (15/129)	1.83 [0.77–4.32]	0.17	
*High (5 items)*	31.5% (110/349)	90.0% (99/110)	10.0% (11/110)	1.57 [0.63–3.90]	0.33	
**Hungry in last 4 weeks**						
*No*	81.1% (283/349)	90.8% (257/283)	9.2% (26/283)	—		
*Yes*	18.9% (66/349)	89.4% (59/66)	10.6% (7/66)	1.15 [0.52–2.54]	0.722	
**Location**						
*Site 1*	51.0% (178/349)	87.1% (155/178)	12.9% (23/178)	—		
*Site 2*	49.0% (171/349)	94.2% (161/171)	5.8% (10/171)	0.45 [0.22–0.92]	**0.029**	
**DREAMS enrollee**						
*No*	62.8% (219/349)	96.3% (211/219)	3.7% (8/219)	—		
*Yes*	37.2% (130/349)	80.8% (105/130)	19.2% (25/130)	5.26 [2.45–11.3]	**< 0.001**	
***Cognitive factors***						
**Self-reported Anxiety & Depression Symptoms**						
*Normal*	63.3% (221/349)	90.5% (200/221)	9.5% (21/221)	—		
*Mild/Moderate/Severe*	36.7% (128/349)	90.6% (116/128)	9.4% (12/128)	0.99 [0.50–1.94]	0.969	
**Comprehensive HIV Knowledge**						
*No*	40.1% (140/349)	92.9% (130/140)	7.1% (10/140)	—		
*Yes*	59.9% (209/349)	89.0% (186/209)	11.0% (23/209)	1.54 [0.76–3.14]	0.233	
***Sexual Behaviors, Experiences, and/or Circumstances***						
**At risk for HIV (> = 1 sex-related factor)**						
*No*	1.1% (4/349)	100.0% (4/4)	0.0% (0/4)	‡		
*Yes*	98.9% (345/349)	90.4% (312/345)	9.6% (33/345)	‡	‡	
**Inconsistent condom use**						
*No*	25.2% (88/349)	81.8% (72/88)	18.2% (16/88)	—		—
*Yes*	74.8% (261/349)	93.5% (244/261)	6.5% (17/261)	0.36 [0.19–0.68]	**0.002**	0.77 [0.37–1.61]
**STI symptoms**^**a**^						
*No*	80.8% (282/349)	91.8% (259/282)	8.2% (23/282)	—		—
*Yes*	19.2% (67/349)	85.1% (57/67)	14.9% (10/67)	1.83 [0.92–3.66]	0.087	1.38 [0.71–2.68]
**Age-disparate sex**						
*No*	42.2% (145/344)	91.7% (133/145)	8.3% (12/145)	—		
*Yes*	57.8% (199/344)	89.9% (179/199)	10.1% (20/199)	1.21 [0.61–2.40]	0.577	
**Inter-generational sex**						
*No*	89.2% (307/344)	90.2% (277/307)	9.8% (30/307)	—		
*Yes*	10.8% (37/344)	94.6% (35/37)	5.4% (2/37)	0.55 [0.14–2.22]	0.404	
**Transactional sex**						
*No*	96.0% (335/349)	90.7% (304/335)	9.3% (31/335)	—		
*Yes*	4.0% (14/349)	85.7% (12/14)	14.3% (2/14)	1.54 [0.41–5.81]	0.521	
**Multiple sexual partners**						
*No*	86.2% (301/349)	90.7% (273/301)	9.3% (28/301)	—		
*Yes*	13.8% (48/349)	89.6% (43/48)	10.4% (5/48)	1.12 [0.45–2.76]	0.806	
**Multiple sexual partners & Inconsistent condom use**^**a**^						
*No*	91.4% (319/349)	90.3% (288/319)	9.7% (31/319)	—		
*Yes*	8.6% (30/349)	93.3% (28/30)	6.7% (2/30)	0.69 [0.17–2.73]	0.593	
**Alcohol use before sex**						
*No*	73.4% (256/349)	90.6% (232/256)	9.4% (24/256)	—		
*Yes*	26.6% (93/349)	90.3% (84/93)	9.7% (9/93)	1.03 [0.50–2.14]	0.932	
**Partner(s) does not live in community**						
*No*	68.2% (238/349)	94.1% (224/238)	5.9% (14/238)	—		—
*Yes*	31.8% (111/349)	82.9% (92/111)	17.1% (19/111)	2.91 [1.52–5.59]	**0.001**	1.49 [0.73–3.04]
**Partner(s) had other partners**						
*No*	65.9% (230/349)	92.2% (212/230)	7.8% (18/230)	—		
*Don't know*	26.4% (92/349)	88.0% (81/92)	12.0% (11/92)	1.53 [0.75–3.11]	0.242	
*Yes*	7.7% (27/349)	85.2% (23/27)	14.8% (4/27)	1.89 [0.69–5.18]	0.214	
**Does not know partner(s) HIV status**						
*No*	81.7% (285/349)	90.5% (258/285)	9.5% (27/285)	—		
*Yes*	18.3% (64/349)	90.6% (58/64)	9.4% (6/64)	0.99 [0.43–2.30]	0.981	
**Partner(s) likely exposed to HIV**^**a**^						
*No*	79.4% (277/349)	96.8% (268/277)	3.2% (9/277)	—		—
*Don't know*	12.3% (43/349)	79.1% (34/43)	20.9% (9/43)	6.44 [2.71–15.3]	**< 0.001**	**6.22 [2.71–14.20]**
*Yes*	8.3% (29/349)	48.3% (14/29)	51.7% (15/29)	15.9 [7.65–33.1]	**< 0.001**	**10.70 [5.07–22.70]**
**Non-partner sexual violence survivorship**						
*No*	87.1% (304/349)	92.4% (281/304)	7.6% (23/304)	—		—
*Yes*	12.9% (45/349)	77.8% (35/45)	22.2% (10/45)	2.94 [1.50–5.76]	**0.002**	**2.04 [1.04–4.00]**
**Physical and/or sexual violence survivorship**						
*No*	54.7% (191/349)	92.1% (176/191)	7.9% (15/191)	—		
*Yes*	45.3% (158/349)	88.6% (140/158)	11.4% (18/158)	1.45 [0.76–2.78]	0.263	
**Post-exposure prophylaxis use**^**a**^						
*No*	97.7% (340/348)	90.3% (307/340)	9.7% (33/340)	‡		
*Yes*	2.3% (8/348)	100.0% (8/8)	0.0% (0/8)	‡	‡	

Bolded aPR [95% CI] indicates *p* ≤ 0.05

*PR* Prevalence Ratio, *CI* Confidence Interval, *APR* Adjusted Prevalence Ratio

^a^ Oral pre-exposure prophylaxis eligibility criterion

^‡^ Regression model not fitted due to small cell sizes

### Inter-country correlate comparisons

**Fig. 1:**
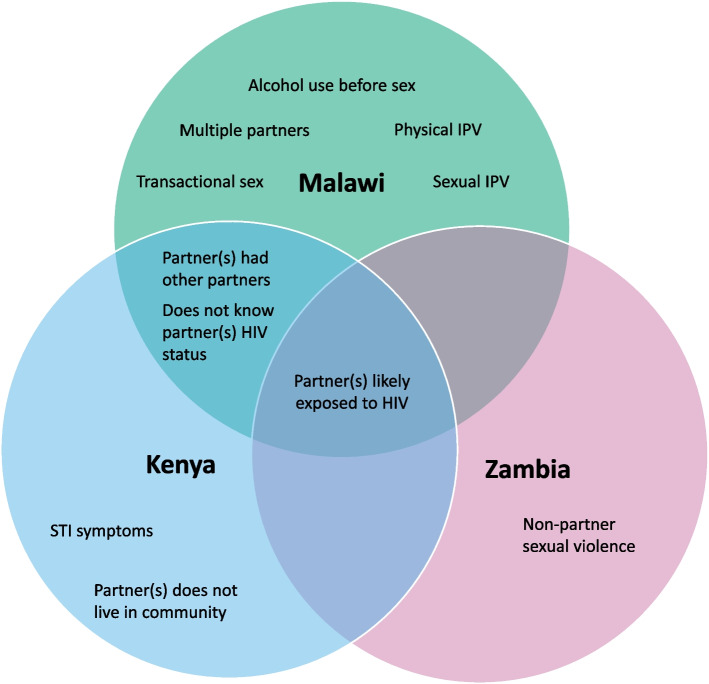
Positive correlates of self-appraised likely HIV exposures among AGYW

Figure 1 summarizes the correlates within and across countries. Partner(s)’ likely HIV exposure emerged as a correlate in all three countries. In Kenya and Malawi, partner(s)’ other partners and unknown HIV status were also correlates. Otherwise, Malawi had the most unique associations, encompassing transactional sex, multiple partnerships, alcohol use, and physical and sexual IPV. Kenya’s unique associations included STI symptoms and partner(s)’ non-community residence, whereas non-partner sexual violence was Zambia’s one unique correlate.

### Intersections between PrEP eligibility and perceived HIV risk in AGYW with HIV risk

**Fig. 2:**
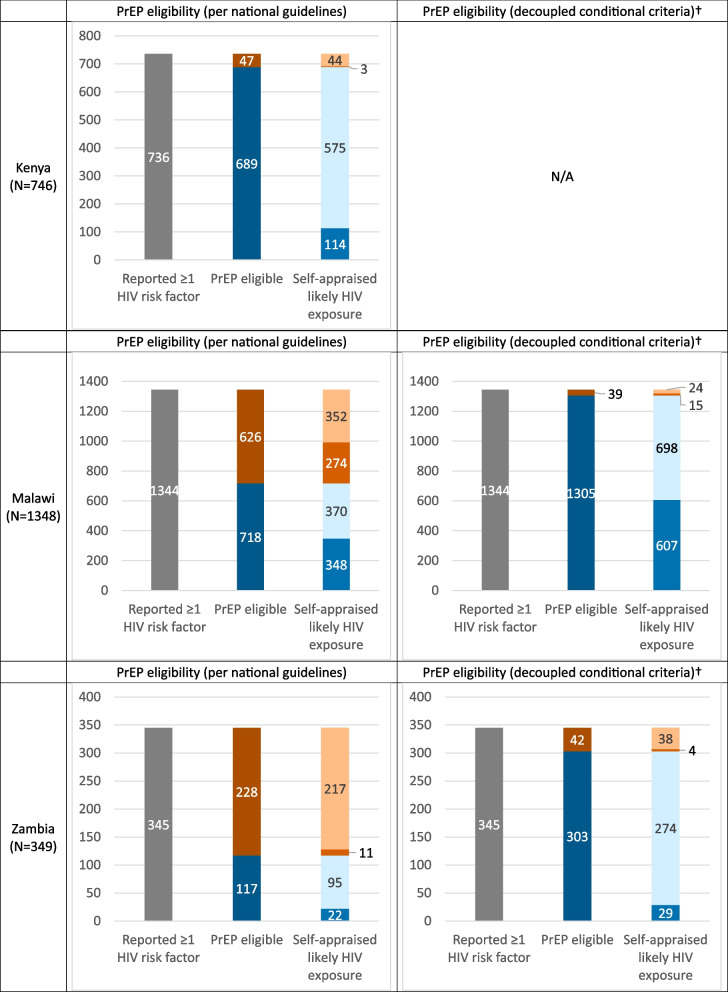
Distribution of HIV risk, PrEP eligibility, and Self-appraised likely HIV exposures † Malawi’s and Zambia’s national PrEP guidelines each included conditional criteria (Malawi: ever-pregnant + age-disparate relationships, Zambia: inconsistent condom use + multiple partnerships). This PrEP eligibility examination decouples these conditional criteria and treats each factor as their own criterion N/A = Not applicable because Kenya had no conditional PrEP criteria in their national guidelines In the PrEP eligibility column, Dark Blue indicates PrEP-eligible, and Dark Orange represents PrEP-ineligible. In the self-appraised HIV exposure column, Blue highlights self-reported likely HIV exposures among PrEP-eligible AGYW, whereas Light Blue represents unlikely exposures in this same group; Orange indicates likely HIV exposures among PrEP-ineligible AGYW, while Light Orange reflects unlikely exposures in the same classification

Figure 2 presents the overlapping frequency of PrEP eligibility and self-appraised likely HIV exposures among AGYW at risk for HIV acquisition (≥ 1 sexual-related risk factor). Using national guidelines, PrEP eligibility captured 93.6% (689/736) of at-risk AGYW in Kenya but only 53.4% (718/1344) in Malawi and 33.9% (117/345) in Zambia; however, these proportions increased to 97.1% and 87.8% in Malawi and Zambia, respectively, after decoupling their conditional PrEP eligibility criteria.

Self-appraised likely HIV exposures were relatively low among PrEP-eligible AGYW (Kenya: 16.5%, Malawi: 48.5%, Zambia: 18.8%). Also, few PrEP-ineligible AGYW reported likely HIV exposures (Kenya: 6.4%, Malawi: 43.7%, Zambia: 4.8%). While Malawi’s and Zambia’s decoupled PrEP criteria did not proportionally increase self-appraised HIV exposures among PrEP-eligible AGYW (46.5% and 9.6%, respectively), the number of AGYW falling within these categories respectively increased by 74.4% and 31.8%.

## Discussion

In this study with sexually active AGYW in Kenya, Malawi, and Zambia, self-reported likely HIV exposures were uncommon amid ubiquitous HIV risk (i.e., reporting ≥ 1 HIV risk factor). Correlates of likely HIV exposure primarily related to factors beyond respondents’ locus of control, spanning partner(s)’ likely HIV exposure (all countries), other partners (Kenya, Malawi), unknown status (Kenya, Malawi), non-community residence (Kenya); physical and sexual IPV (Malawi); and non-partner sexual violence (Zambia). Other correlates included STI symptoms in Kenya and transactional sex, multiple partners, and pre-coital alcohol use in Malawi. Exploring overlaps between HIV risk perception and PrEP eligibility highlighted that Kenya’s criteria captured most at-risk AGYW. Decoupling conditional criteria in Malawi’s (age-disparate sex + ever-pregnant) and Zambia’s (multiple partners + condomless sex) guidelines could improve risk identification. Self-reported likely HIV exposures were low across PrEP-eligibility groupings, but decoupling Malawi’s criteria could increase PrEP engagement opportunities. Without improving AGYW’s actual-perceived risk disconnect, HIV prevention services and interventions will remain underutilized.

Partnership-related factors, primarily in Kenya and Malawi, influenced AGYW’s HIV risk perception most, specifically partner(s)’ likely HIV exposure, having other partners, unknown HIV status, and non-community residence. Other studies also found partners’ behaviors and characteristics influence AGYW’s HIV risk perception [45–50]. Our findings could imply AGYW do not know partner(s) HIV status and, thus, assume partner(s) are likely exposed to HIV due to other relationships. Qualitative data from ESA supports this hypothesis, as the studies also found partner distrust increases AGYW’s HIV risk perception [46, 51]. These results also reflect the magnitude of disempowerment experienced by AGYW in gender-inequitable environments, where male partners often engage in HIV-associated behaviors, control condom negotiations, and influence HIV prevention decision-making [52–56]. These points also highlight the complexity of examining AGYW’s HIV risk behaviors and perceptions in these settings and circumstances, as it is difficult to gauge which behaviors AGYW willingly and consciously initiated versus those being byproducts of power differentials [57, 58]. More robust questions and deeper probing into behavioral reasoning (e.g., who made the decision to not use the condom?) may better elucidate the intricate relationships affecting behaviors and perceptions.

Concordant with HIV risk research, AGYW’s survivorship of IPV and non-partner violence also affected HIV risk perceptions [30, 59, 60]. In Malawi, physical and sexual IPV increased AGYW’s risk perception. Our findings conflict with another Malawi study by Price et al*.* yet aligns with findings in other settings, including our prior Kenyan study and qualitative research from southern Africa, Kenya, and Zambia [30, 46, 57, 61, 62]. Since we ostensibly offer first evidence of this possible connection in Malawi, mixed-methods inquiries are needed to better illuminate breadth, depth, and perspectives. Non-partner sexual violence affected Zambian AGYW’s self-appraised HIV risk, concordant with our prior research [30]. However, we could not corroborate with other evidence because non-partner sexual violence remains understudied [63]. Zambia’s urban setting could have affected AGYW’s risk for, and perception of, non-partner sexual violence, as qualitative data from other peri-urban environments show AGYW avoid bars, traveling after dark/before sunrise, and cramped corridors to mitigate sexual violence risk [64]. The perpetrator might also change AGYW’s view of violence and HIV risk: Ugandan AGYW viewed sexual violence within relationships as an expectation, while stranger-perpetrated violence held a more serious connotation [65].

The remaining associative factors in Malawi—transactional sex, multiple partnerships, pre-coital alcohol use—and Kenya, STI symptoms, also aligned with epidemiologic data [61, 66–69]. These findings add new insights to Malawi’s evidence base, as in Price et al*.*, all three factors were associated with HIV status but only transactional sex was associated with HIV risk perception [61]. Additionally, while these factors appear more intrinsic to AGYW, Malawi’s low-income economy means these behaviors could be symptomatic of structural and financial inequalities, especially since school leaving limits earning potential and increases financial dependency [70–73]. We cannot ascertain AGYW’s autonomy to participate in these behaviors, as they might have been survival tactics or coerced upon them by older men, the typical providers in transactional relationships [53, 74–77]. For Kenya, perhaps STI symptoms prompted AGYW to re-evaluate recent behaviors, increasing the proximity and perceptions of HIV risk [78, 79]. Our findings, both statistically significant and null, suggest further quantitatively and qualitatively examining AGYW’s power in sexual decision-making, as well as their motivations for, and perceptions of, engaging in HIV-associated behaviors.

We found self-appraised HIV exposures were low in PrEP-eligible and -ineligible AGYW, suggesting most PrEP-eligible respondents are unlikely to seek out PrEP [17–19]. Currently, Kenya, Malawi, and Zambia do not consider direct PrEP requests as an indication for eligibility [35–39]. Based on the number of self-appraised HIV exposures among PrEP-ineligible AGYW, adding self-selection to Kenya’s and Zambia’s national guidelines might not meaningfully increase PrEP engagement [17–19]. Conversely, this exclusion yielded numerous missed opportunities in Malawi. PrEP self-selection is the behavioral manifestation of perceived risk, and other countries are already incorporating it into their eligibility assessments [17, 80]. Although it may only lead to marginal increases in some settings, Kenya, Malawi, and Zambia should consider making self-selection an eligibility indication in their guidelines. In addition to streamlining service delivery by removing the need to screen self-selectors, this approach may also expand PrEP reach, coverage, and impact, especially when LA-PrEP methods become widely available.

Our analysis supports that criteria-based eligibility might prevent sexually active AGYW from receiving the care and/or interventions necessary to reduce their HIV risk. By decoupling their conditional PrEP criteria, Malawi and Zambia could correctly identify > 85% of at-risk AGYW and potentially increase PrEP interactions by ~ 70% and ~ 30%, respectively [37–39]. These findings underscore the World Health Organization's guidance that eligibility criteria should not create undue barriers to accessing PrEP [81, 82]. At minimum, Malawi and Zambia should relax their conditional criteria to better capture at-risk AGYW and potentially increase PrEP initiation and use. Alternatively, universal counseling might be an optimal strategy for PrEP provision, as it synergizes the benefits of self-selection and criteria adjustments. Recent trial evidence found criteria-based PrEP offers were not superior to universal PrEP counseling among pregnant women. Universal counseling might also increase initiations in at-risk PrEP-ineligible AGYW, overcome AGYW’s low PrEP knowledge, and remedy inefficient or biased outreach, consultation, and counseling processes [83–86]. Increasing the effectiveness of this optimized counseling approach will require concurrent efforts to establish provider–client trust, reduce stigma enacted by healthcare personnel, and simplify medicalized delivery pathways [87–91]. To meaningfully increase PrEP reach and coverage, these service-level changes must be complemented with community-based activities to create PrEP demand in AGYW, potentially through awareness campaigns and peer outreach [92, 93]. Meeting AGYW where they are with age-, context-, and risk-appropriate information may improve their perspective of PrEP services and increase the likelihood they will seek out, access, and use them.

In ESA, PrEP availability and accessibility has increased significantly over the past decade due to national governments partnering with bi- and multilateral partners, like the United States President’s Emergency Plan for AIDS Relief (PEPFAR) and Global Fund to Fight AIDS, Tuberculosis and Malaria (Global Fund) [94, 95]. Through investments and collaborations, PEPFAR and Global Fund have supported national governments to introduce and roll out PrEP methods, nationally and locally. Consequently, total global PrEP initiations are highest in PEPFAR- and Global Fund-supported countries—including Kenya (~ 536k), Malawi (~ 158k), and Zambia (~ 809k) [96]. For AGYW, DREAMS served as an important vehicle for PrEP expansion, increasing new AGYW initiations from almost 8k in 2017 to approximately 341k in 2022, with nearly half occurring after 2020 [97, 98]. Despite these achievements, almost all PrEP-to-need ratio estimates for 2020 were < 1 in DREAMS countries, underscoring current implementation approaches to reach AGYW at risk for HIV acquisition are suboptimal [98]. Findings and recommendations presented within this article could partially rectify this issue by increasing AGYW’s PrEP accessibility within Kenya, Malawi, and Zambia, nationally and as part of DREAMS NextGen implementation [97].

### Limitations

Our study has some limitations. We could not fully measure physical and sexual IPV in Kenya and Zambia due to questionnaire limitations: our null findings might misrepresent actual associations. Zambia’s models are possibly overfitted given the small number of events. Surveys did not measure or fully capture serodifferent relationships, injection drug use, PEP use (Kenya), and violence (Kenya, Zambia); thus, the number of PrEP-eligible AGYW might be underrepresented. Also, surveys used a 12-month recall period for most measures, while guidelines use a 6-month period, potentially inflating estimates of PrEP-eligible AGYW. Since all the data were self-reported, including HIV status, our data were potentially affected by social desirability, which could have attenuated effects and associations.

## Conclusions

Sexually active AGYW in Kenya, Malawi, and Zambia are at increased risk for HIV. HIV risk perception could motivate AGYW to seek out, access, and use HIV prevention services and interventions, but AGYW’s low HIV risk perception often contradicts their actual HIV risk. In examining correlates of AGYW’s HIV risk perception, we discovered extrinsic factors—partner(s)’ likely HIV exposure, other partners, unknown HIV status, and non-community residence; and physical and sexual violence from partners and non-partners—primarily increased AGYW’s HIV risk perception. Transactional sex, multiple partnerships, and pre-coital alcohol use also increased Malawian AGYW’s risk perception, as did STI symptoms in Kenyan AGYW. Complementary analyses of overlaps between PrEP eligibility and risk perception highlighted self-appraised HIV exposures were low across PrEP-eligible and -ineligible categorizations. To meaningfully increase access and use of HIV prevention services and interventions, improvements in AGYW’s HIV risk appraisal, identification, and perception are needed; deeper examination of the interplay between agency, behaviors, and perceptions would advance this goal. Otherwise, their current suboptimal utilization of HIV prevention services and methods will persist.

## Supplementary Information

Supplementary Material 1.

Supplementary Material 2.

## Data Availability

The underlying data used in these analyses are publicly available via Dataverse.
