# Supplementary material for: Examining concordance of sexual-related factors and PrEP eligibility with HIV risk perception among adolescent girls and young women: cross-sectional insights from DREAMS sites in Kenya, Malawi, and Zambia
Source: BMC Public Health. 2024 Oct 12;24:2793. doi: 10.1186/s12889-024-20276-4 (PMC11470662; doi:10.1186/s12889-024-20276-4)
Supplement: Supplementary file 1 — Supplementary Material 1. [file 12889_2024_20276_MOESM1_ESM.docx]

**Supplemental Material 1. Study measures**

| **Measure** | **Variable Type** | **Definition** |
| --- | --- | --- |
| **Dependent variable** | | |
| **Likely exposed to HIV** | Binary | Regarded their exposure to HIV as very likely or somewhat likely versus unlikely or not at all |
| ***Sociodemographic factors*** | | |
| **Age (categorical)** | Binary | Dichotomized continuous age to differentiate between adolescent girls (aged 15-19 years) and young women (aged 20-24 years) |
| **Ever Pregnant** | Binary | Currently or previously pregnant |
| **Orphaned** | Binary | At least 1 parent is deceased |
| **Out of School** | Binary | Not currently in school |
| **Currently Employed** | Binary | Compares employed individuals versus those who are only students and/or unemployed |
| **Married** | Binary | Currently married or living with a man as if married |
| **Socioeconomic Status** | Categorical / Binary | Using a count of household assets (piped water, flushed toilet, private toilet, finished floors, metal/tiled roof), a trichotomous variable was created using the median and interquartile range: Low = 25th percentile or lower, Medium = Median, and High = 75th percentile or higher. For Kenya, the median and 75th percentile were both 3, leaving few respondents in the High category. Thus, for the Kenya-specific analyses, a binary variable (Low vs. Medium or High) is used. |
| **Hungry in last 4 weeks** | Binary | Respondent or household member went to sleep without eating due to lack of food, dichotomized to represent ever (often, sometimes, rarely) versus never. |
| **Location** | Binary | Differentiates study sites |
| **DREAMS enrollment status** | Binary | Pre-intervention enrollment status into DREAMS. In Malawi, all the respondents were DREAMS enrollees |
| ***Cognitive factors*** | | |
| **Self-reported Anxiety & Depression Symptoms** | Categorical/Binary | Using the PHQ-4, the summed scores of the four measures were categorized to represent Normal (<3 score), Mild (3-5 score), Moderate (6-8 score) and Severe (>8 score). Due to limited variability in all countries regarding the Moderate and Severe categories, all analyses used a binary variable to measure Mild, Moderate, or Severe vs. Normal symptoms. |
| **Comprehensive HIV Knowledge** | Binary | We used the Demographic and Health Survey's measure comprehensive HIV knowledge. These measure included knowing two primary prevention methods that reduce the chance of getting HIV (1. use of condoms, 2. having just one uninfected partner), understanding that healthy-looking people can have HIV, and rejecting two common local misconceptions regarding HIV/AIDS transmission or prevention (HIV cannot be transmitted by 4. mosquito bites or 5. sharing food). We created a binary variable to represent respondents who answered all five questions correctly versus those who did not. |
| ***Sexual Behaviors, Experiences, and/or Circumstances*** | | |
| **At risk for HIV (>1 sex-related factor)** | Binary | Reported at least one sexual behavior, experience, and/or circumstance associated with epidemiologic HIV risk |
| **Condoms inconsistently used** | Binary | In the last 12 months, inconsistently used condoms with their primary and/or secondary partner or did not use a condom at last sex with primary and/or secondary partner |
| **STI symptoms** | Binary | Experienced STI symptoms (genital ulcers, vaginal discharge, painful urination, or genital warts) in the last 6 months |
| **Age-disparate sex** | Binary | Primary or secondary partner was 5 or more years older |
| **Inter-generational sex** | Binary | Primary or secondary partner was 10 or more years older |
| **Transactional sex** | Binary | In the last 12 months, engaged in sex with a stranger or casual partner for financial or material support (e.g., money for children or family, somewhere to stay, transportation, cell phone, etc.) |
| **Multiple sexual partners** | Binary | Reported more than 1 sexual partner in the last 12 months or in the partner grid, stated they had a secondary partner |
| **Alcohol used before sex** | Binary | Respondent or primary and/or secondary partner used alcohol before sex |
| **Partner(s) does not live in community** | Binary | Primary or secondary partner did not regularly live in the community. |
| **Partner(s) had other partners** | Binary | Respondent said that in the last 12 months, their primary and/or secondary partner had other partners, they did not know, or their primary and/or secondary partner did not have other partners. |
| **Does not know partner(s) HIV status** | Binary | Respondent did not affirmatively state they knew the HIV status of their primary or (when applicable) secondary partner. |
| **Partner(s) likely exposed to HIV** | Binary | Respondent stated that the likelihood that their primary and/or secondary partner was exposed to HIV was very likely or somewhat likely vs. don't know vs. unlikely or not at all. |
| **Physical intimate partner violence survivorship** | Binary | In the last 12 months, a current or previous partner physically assaulted the respondent using slaps or thrown objects; pushes or shoves; hits with a fist or other dangerous object; kicks, yanks or pulls, beatings, chokings, or burnings; or threats with a gun, knife, or other weapon. Only reported for Malawi due to the reasons noted below. |
| **Sexual intimate partner violence survivorship** | Binary | In the last 12 months, a current or previous partner used physical coercion, threats and intimation, or force to perpetrate unwanted sex or other sexual acts upon the respondent. Only reported for Malawi due to the reasons noted below. |
| **Non-partner sexual violence survivorship** | Binary | In the last 12 months, a person other than a current or previous partner forced sex upon the respondent using persuasion, alcohol- or drug-induced impairment, or the assistance of one or more men. Asked of all AGYW in all three countries. |
| **Physical and/or sexual violence survivorship** | Binary | Composite variable measuring survivorship of physical or sexual intimate partner violence, sexual violence by a non-partner, or seeking post-violence care in the last 12 months.  Due to a skip logic, the intimate partner violence questions were not asked of all sexually active AGYW in Kenya (26.4% [197/768] not asked) and Zambia (23.5% [82/349] not asked). To construct this variable in these countries, we added affirmative responses regarding post-violence care and physical or sexual intimate partner violence to the non-partner sexual violence variable, which was asked of all AGYW. After these transformations, 142 and 62 AGYW in Kenya and Zambia, respectively, with missing data for the intimate partner violence questions had "No" responses for both non-partner sexual violence and seeking out post-violence care. As an exploratory exercise, we kept these respondents coded as "No," acknowledging that this coding would bias toward the null. Thus, if we do observe an association in these countries, it could be potentially stronger than measured in our samples. In Malawi, these questions were asked of all sexually active AGYW and, thus, no additional assumptions are needed. |
| **Age-disparate sex & Ever-pregnant** | Binary | Composite variable combining positive indications of age-disparate sex and ever being pregnant.  Malawi-specific risk factor due to its inclusion in the risk assessment for oral PrEP eligibility, as defined by Malawi’s national PrEP guidelines |
| **Multiple sexual partners & Inconsistent condom use** | Binary | Composite variable combining positive indications of multiple sex partners and inconsistent condom use.  Zambia-specific risk factor due to its inclusion in the risk assessment for oral PrEP eligibility, as defined by Zambia’s national PrEP guidelines |
| **Post-exposure prophylaxis use** | Binary | Ever visited a health facility or doctor of any kind to receive post-exposure prophylaxis.   Only measured in Zambia. |
| **PrEP eligible (>1 criteria)** | Binary | This composite variable captures the presence of at least one oral PrEP indication, representing the PrEP eligibility of the respondent.  In Kenya, measured criteria included multiple partnerships, inconsistent condom use, don’t know partner(s) status, partner(s) likely HIV exposure, STI symptoms, alcohol use before sex, transactional sex, and survivorship from any form of violence.   In Malawi, measured criteria comprised STI symptoms, transactional sex, and ever-pregnant + age-disparate relationships.   In Zambia, measured criteria encompassed inconsistent condom use + multiple partnerships, partner(s) likely HIV exposure, STI symptoms, and post-exposure prophylaxis use.  Due to questionnaire limitations, we could not examine sero-different partnership characteristics, dynamics, and factors (all countries). Sharing of injection paraphernalia could not be assessed in Kenya and Zambia, and post-exposure prophylaxis use could not be examined in Kenya. |
