# Supplementary material for: Examining concordance of sexual-related factors and PrEP eligibility with HIV risk perception among adolescent girls and young women: cross-sectional insights from DREAMS sites in Kenya, Malawi, and Zambia
Source: BMC Public Health. 2024 Oct 12;24:2793. doi: 10.1186/s12889-024-20276-4 (PMC11470662; doi:10.1186/s12889-024-20276-4)
Supplement: Supplementary file 2 — Supplementary Material 2. [file 12889_2024_20276_MOESM2_ESM.pptx]

## Slide 1
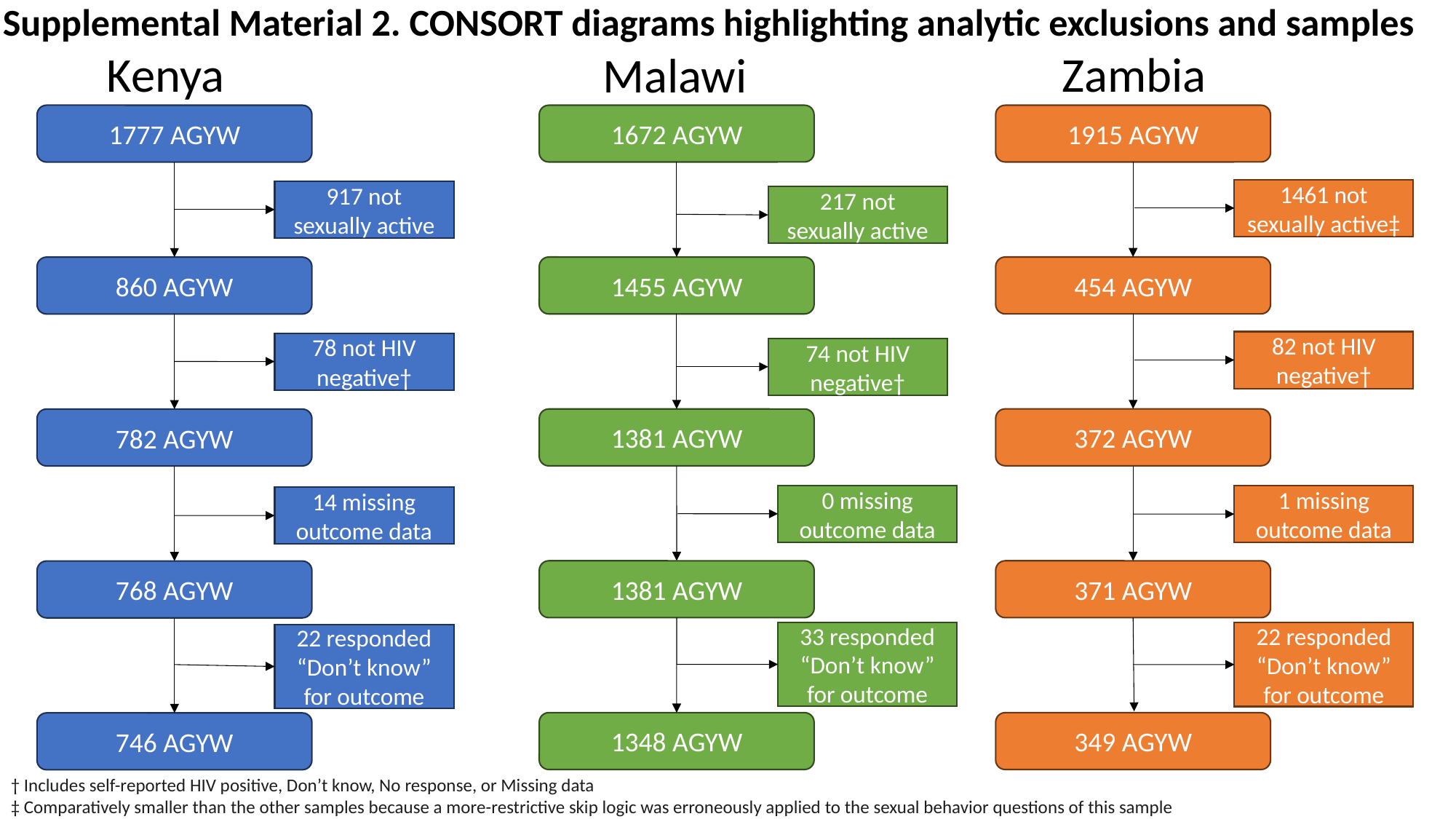

Supplemental Material 2. CONSORT diagrams highlighting analytic exclusions and samples
Zambia
Kenya
Malawi
1915 AGYW
1672 AGYW
1777 AGYW
1461 not sexually active‡
917 not sexually active
217 not sexually active
454 AGYW
1455 AGYW
860 AGYW
82 not HIV negative†
78 not HIV negative†
74 not HIV negative†
372 AGYW
1381 AGYW
782 AGYW
0 missing outcome data
1 missing outcome data
14 missing outcome data
1381 AGYW
371 AGYW
768 AGYW
33 responded “Don’t know” for outcome
22 responded “Don’t know” for outcome
22 responded “Don’t know” for outcome
1348 AGYW
349 AGYW
746 AGYW
† Includes self-reported HIV positive, Don’t know, No response, or Missing data
‡ Comparatively smaller than the other samples because a more-restrictive skip logic was erroneously applied to the sexual behavior questions of this sample
